# A Case Report on Serotonin Syndrome in a Patient With Parkinson’s Disease: Diagnostic and Management Challenges

**DOI:** 10.7759/cureus.36780

**Published:** 2023-03-28

**Authors:** Marko Nemet, Ana Andrijević, Đorđe Nedeljkov, Vladimir Andrić, Srđan Gavrilović

**Affiliations:** 1 Internal Medicine, University of Novi Sad, Novi Sad, SRB; 2 Intenisve Care Unit, Institute for Pulmonary Diseases of Vojvodina, Sremska Kamenica, SRB; 3 Intensive Care Unit, Institute for Pulmonary Diseases of Vojvodina, Sremska Kamenica, SRB; 4 Emergency Medicine, University of Novi Sad, Novi Sad, SRB

**Keywords:** snri, duloxetine, rasagiline, mao inhibitors, parkinson's disease, malignant syndrome, serotonin syndrome (ss)

## Abstract

Patients with Parkinson's disease are often at risk of polypharmacy, which can lead to serious medication side effects and interactions. Serotonin syndrome (SS) can develop in this patient population due to a possible drug-drug interaction between antidepressants and antiparkinson drugs with serotoninergic activity. On the other hand, these patients are also at risk of malignant syndrome (MS) secondary to dopaminergic medication withdrawal. In this case report, we present a 71-year-old female with Parkinson's disease who developed symptoms suggestive of SS. The patient was admitted to the medical intensive care unit at the Institute for Pulmonary Diseases of Vojvodina in the Republic of Serbia due to impaired consciousness and a previously witnessed cardiorespiratory arrest. Her chronic antiparkinson medication regimen consisted of levodopa, benserazide, entacapone, ropinirole, and rasagiline. Furthermore, she had been prescribed duloxetine for a remote history of depression, which she had only been taking intermittently. Several days before admission, however, the patient started taking duloxetine again due to low mood. Upon admission, laboratory tests revealed leukocytosis with neutrophilia, elevated C-reactive protein, procalcitonin, lactate, urea, and creatinine. Serum creatine kinase (CK) levels were also elevated at 1250 U/L. Six hours after admission to the ICU, the patient developed hyperthermia, hyperreflexia, spontaneous myoclonus, and tremors. Her CK levels continued to rise, reaching 6900 U/L, and her renal function worsened. Due to the possibility of either SS or MS, external cooling measures with frozen gel packs were administered, resulting in the patient's stabilization over a few hours. Further, serotoninergic medication (rasagiline and duloxetine) was discontinued. On the fifth day of hospitalization, a head CT showed signs of cytotoxic edema. On the 11th day, the patient became hemodynamically unstable and passed away despite all adequate resuscitative measures.

The purpose of this case report is to raise awareness of possible SS in patients taking monoamine oxidase-B (MAO-B) inhibitors such as rasagiline. Clinicians should have a high index of suspicion for this complication, especially in patients who are treated for comorbid depression with serotoninergic drugs. Furthermore, we emphasize the importance of correctly differentiating SS from MS, which are both risks for patients with Parkinson's disease. A correct approach to these patients is of utmost importance for adequate management and optimal outcomes.

## Introduction

Serotonin syndrome (SS) is a potentially life-threatening condition that occurs secondary to serotonergic overactivity in the central and peripheral nervous system. The main culprits for this condition are serotoninergic drugs [1].

The classic triad of symptoms is neuromuscular excitability, autonomic dysfunction, and altered mental status. Neuromuscular hyperactivity can present as hypertonia, hyperreflexia, myoclonus, and tremor. Autonomic dysfunction can range from diaphoresis, tachycardia, hyperthermia, and hypertension to hemodynamic instability and hypotension [2]. Finally, changes in mental status include anxiety, agitation, confusion, and delirium [3]. In severe SS, multiorgan failure can develop, manifesting as seizures, acute kidney injury, rhabdomyolysis, disseminated intravascular coagulation, and acute respiratory distress syndrome. In these cases, patients are at increased risk of lethal outcomes [4].

Serotonin syndrome is considered a diagnosis of exclusion. However, there are available criteria for establishing the diagnosis. The two most commonly used criteria are Sternbach’s criteria and the Hunter serotonin toxicity criteria [5]. These criteria are based solely on the classical clinical findings in SS, such as altered mental status, hyperreflexia, and fever.

Drugs responsible for SS have different mechanisms for increasing serotoninergic transmission. Some of the most common causative agents work as inhibitors of serotonin metabolism such as monoamine oxidase inhibitors (MAOI). Others are inhibitors of serotonin uptake from the synaptic cleft like selective serotonin reuptake inhibitors (SSRIs), serotonin-norepinephrine reuptake inhibitors (SNRIs), and tricyclic antidepressants. While SS can occasionally happen in patients who take a single serotoninergic substance, it is more frequent and severe in cases of a combination of two or more substances that potentiate serotoninergic pathways [1].

Monoamine oxidase inhibitors (MAOI) are recognized as one of the most dangerous drugs that lead to severe SS, especially in combination with other serotoninergic medications. There are two classes of MAOI, MAO-A inhibitors and MAO-B inhibitors [6]. The MAO-A are inhibitors of the metabolism of serotonin and norepinephrine and are the type of MAOI commonly referred to as responsible for SS. On the other hand, MAO-B inhibitors such as selegiline and rasagiline, are selective inhibitors of dopamine metabolism in the central nervous system [6]. The MAO-B inhibitors are used as adjunct drugs in Parkinson's disease as they increase central dopamine levels. Even though MAO-B inhibitors are not designed to affect serotonin pathways, these drugs can lose their selectivity in sufficiently high doses. This could lead to serotoninergic effects [6]. As patients with Parkinson's often develop depression and are treated with antidepressants [6], there is a concern for a possible drug-drug interaction between classic antidepressants and MAO-B inhibitors that could lead to SS.

This is a case report of a Parkinson's disease patient who was treated for SS at the Institute for Pulmonary Diseases in Vojvodina, Republic of Serbia. Our aim in presenting this case analysis is to raise awareness of the potential for SS in patients taking MAO-B inhibitors like rasagiline. Additionally, we discuss the importance of distinguishing SS from malignant syndrome (MS), which is also a risk in patients with Parkinson's.

## Case presentation

A 71-year-old Caucasian female was admitted to the medical intensive care unit (MICU) due to impaired consciousness and previously witnessed cardiorespiratory arrest. She was initially found unresponsive and apneic by paramedics in her apartment. After being successfully resuscitated due to pulseless electrical activity, she was admitted to the tertiary university hospital: the Institute for Pulmonary Diseases of Vojvodina in the Republic of Serbia.

Medical history obtained from her son revealed that the patient had Parkinson's disease for seven years. As part of her medication regimen, she was taking several doses of levodopa (700mg/day in five doses), benserazide (175mg/day in five doses), entacapone (400mg/day in two doses), ropinirole (12mg/day in two doses), and rasagiline (1mg/day). Furthermore, she had a remote history of depression that was treated with duloxetine, which she was only taking intermittently. Several days before admission, due to low mood, the patient started taking duloxetine again. The exact dosage regimen of duloxetine was unknown.

Her son further revealed that the patient was febrile for two days before admission. Also, she experienced worsening dyskinetic movements and stiffness, as well as progressive dysphagia. As a result, while taking her medication, the patient choked and lost consciousness.

On admission, the patient’s Glasgow coma scale (GCS) was 7 (eyes 2, verbal 1, motor 4). Continuous sedation was administered as she was on conventional mechanical ventilation with invasive hemodynamic monitoring. Her vital signs were stable except temperature as she was febrile up to 38.8℃. A brief neurological exam showed that pupils were equal, miotic (1.5mm), and reactive and the patient had spontaneous clonic spasms of the upper and lower extremities.

Laboratory values on admission are presented in Table 1. A complete blood count revealed leukocytosis of 11 300/mm^3^ with 90% of neutrophils. In other laboratory findings, C-reactive protein (10mg/L) and procalcitonin (0.09ng/mL) were slightly elevated. Enzymes of hepatocellular necrosis, markers of acute kidney failure, and lactate were elevated as well with a lactate value of 10.85mmol/L. Serum creatine kinase (CK) levels were elevated at 1250U/L. On arterial blood gas analysis, the patient was hypoxemic (partial pressure of oxygen (PaO_2)_, 37.8mmHg) and hypercapnic (partial pressure of arterial carbon dioxide (PaCO_2)_, 68.2mmHg). Bicarbonate levels were 19.5mmol/L and arterial pH was 7.25. These results were consistent with global respiratory failure resulting in respiratory acidosis. This patient also had a high anion gap-metabolic acidosis (AG, 21.5mEq/L) and signs of acute tubular necrosis.

**Table 1:** Laboratory testing on admission and six hours after admission PaO2: Partial pressure of oxygen, PaCO2: Partial pressure of arterial carbon dioxide, HCO3: Bicarbonate, N/A: Not applicable or not available

Variable	Units	On admission	Six hours after admission	Reference range
White blood cells	cells/mm^3^	11 300	8 000	4000-11000
Neutrophils	%	90	80	44-75
C-reactive protein	mg/L	10.0	16.5	<5.0
Procalcitonin	ng/mL	0.99	-	<0.05
Aspartate aminotransferase (AST)	U/L	185.54	786.14	<32.0
Alanine aminotransferase (ALT)	U/L	43.98	338.55	<33.0
Creatinine	mg/dL	1.46	2.28	0.7-1.2
Blood urea nitrogen	mg/dL	37.3	57.5	7.85-22.7
Lactate	mmol/L	10.85	-	0.5-2.2
Creatine kinase	U/L	1250	6900	<170.0
PaO_2_	mmHg	37.8	107.5	80.0-100.0
PaCO_2 _	mmHg	68.2	39.16	35.0-45.0
HCO_3_	mmol/L	19.5	20.2	22.0-29.0
Arterial blood pH	N/A	7.25	7.35	7.35-7.45
Anion gap	mEq/L	21.5	17.6	3.0-10.0

Furthermore, chest X-ray showed no pulmonary infiltrates nor signs of pleural effusion. Urinalysis yielded a high number of bacteria, leucocytes, and erythrocytes. Therefore, a presumptive diagnosis of urinary tract infection was made and antibiotics for urinary tract infection (ceftriaxone and levofloxacin) were initiated. Before administering antibiotics, microbiological samples were taken. 

Six hours after admission to the ICU, the patient became hyperthermic. She also experienced hyperreflexia with spontaneous myoclonus and tremor. Her temperature was 42.3℃ and she was unresponsive to antipyretics. Repeated laboratory values are presented above in Table 1. Her CK levels continued to rise, reaching 6900 U/L, along with a worsening of her renal function. The main differential diagnoses were SS and MS. Therefore, external cooling measures were undertaken with the frozen gel packs. Furthermore, all her serotoninergic medication (rasagiline and duloxetine) were immediately discontinued from her inpatient medication list. Due to aggressive clonic spasms, continuous neuromuscular blockade was initiated. Later during the night, the patient became hemodynamically unstable and norepinephrine (0.2mcg/min) was given. The following morning, her fever subsided and the norepinephrine dose was reduced. On the third day, IV sedation was terminated. However, she remained unconscious with a GCS of 3.

During the following days in the ICU, the patient remained with a GCS of 3 and was febrile and hemodynamically stable on 0.1mcg/kg/min of norepinephrine. Leukocytosis with neutrophilia persisted. Urine and blood cultures came back negative. Acute kidney injury due to presumed acute tubular necrosis was diagnosed with an estimated creatine clearance of 27mL/min. Despite the renal failure, both spontaneous urinary output and blood pH were within normal limits. Thus, she did not require renal replacement therapy.

**Figure 1:**
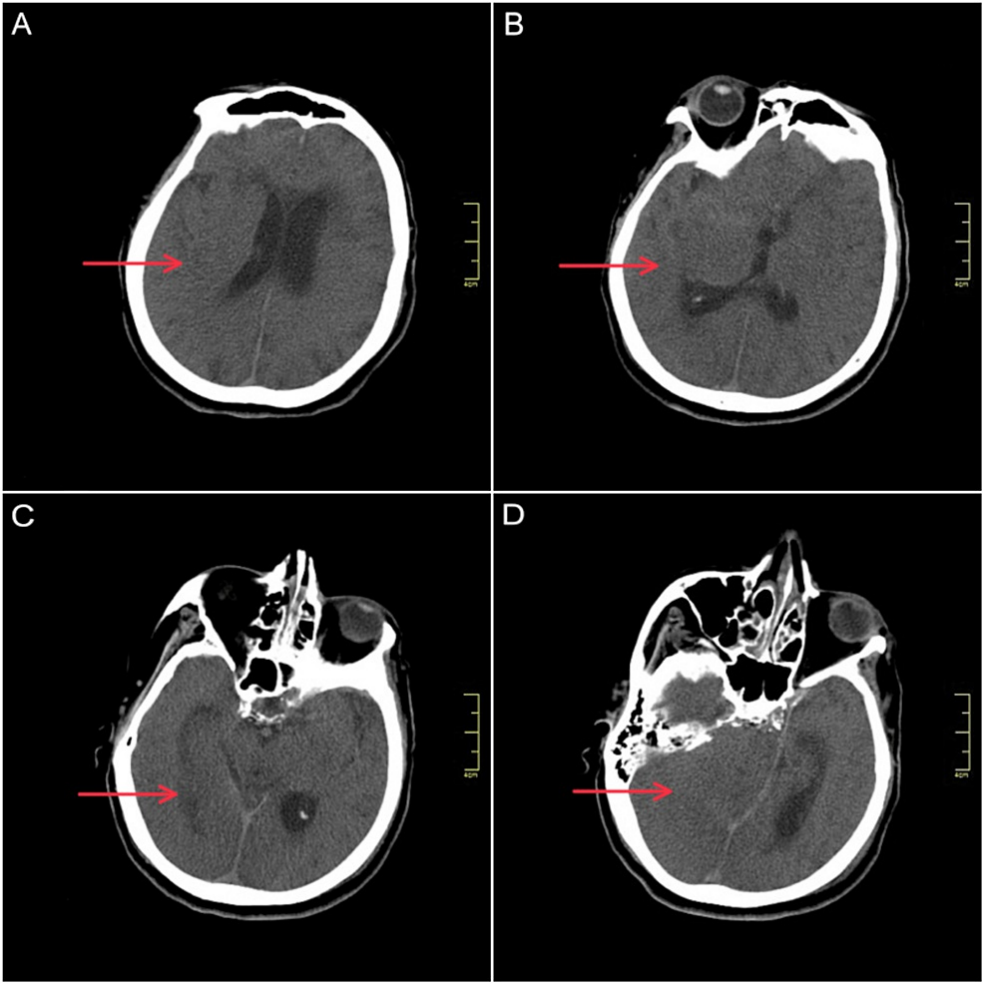
Head CT showing cerebral edema A, B, D: Red arrows show diffuse loss of grey-white differentiation and sulcal effacement; C: The red arrow shows slight dilatation of the temporal horn of the lateral ventricle

On the fifth day of hospitalization, a head CT was performed. It showed signs of cytotoxic edema, with a loss of grey-white differentiation and effaced sulci (Figure 1). On a neurological exam, pupils were unequal and non-reactive. There were no spontaneous respirations and pharyngeal reflex could not be elicited. However, the doll's eye sign was positive. On the seventh day, after a neurology consult, antiparkinson therapy was corrected. Levodopa (700mg/day in five doses) and benserazide (175mg/day in five doses) were continued at the same dose. Ropinirole and entacapone were tapered and discontinued. Mannitol was started for diffuse cerebral edema in the dosage for renal impairment.

During the following days, the patient became afebrile. Nonetheless, the neurological status remained unchanged. On the 11th day, she gradually became hemodynamically unstable without any signs of infection or cardiac decompensation. In the evening of the same day, the patient passed away after all adequate resuscitative measures.

## Discussion

Our report features a case of an adult female patient who developed SS after initiating therapy with duloxetine (an SNRI) while being treated with rasagiline. Our patient satisfied both sets of Sternbach’s and the Hunter serotonin toxicity criteria [5] by experiencing hyperthermia, hyperreflexia, spontaneous myoclonus, and tremor after starting duloxetine while on rasagiline therapy. Even though circulatory collapse can be seen in severe cases of SS, hypertension is more often observed [2]. Our patient required vasopressor support to maintain hemodynamic stability and continuous IV sedation is likely one more contributing factor.

To this date, there is scarce evidence of the SSRI or SNRI-rasagiline combination causing SS. A multicentric retrospective cohort study by Panisset et al. [7] evaluated patients who were treated with rasagiline for Parkinson's disease while taking antidepressants. They found no evidence of SS in these patients. Another, recent study by Hilli et al. [8] found that a combination of rasagiline and escitalopram (SSRI) was well tolerated. Also, no cases of SSwere reported. Nevertheless, there are several case reports of rasagiline and SSRIs leading to SS [9-11]. A summary of these case reports is presented in Table 2. Moreover, there is a case report of a patient developing SS due to rasagiline alone [12]. In this report, the patient was mistakenly treated with 4mg of rasagiline daily, compared to a standard dose of 1mg per day. In our case, the dosage of duloxetine was unknown. While it cannot be excluded that our patient developed SS from high doses of duloxetine only, SS is much more prevalent in patients taking a combination of serotoninergic drugs [1].

**Table 2:** Summary of serotonin syndrome case reports in Parkinson's disease caused by rasagiline-SSRI interaction PD: Parkinson's disease; CK: Creatine kinase; CrCl: Creatinine clearance; BUN: Blood urea nitrogen; AST: Aspartate aminotransferase; ALT: Alanine aminotransferase; sCr: Serum creatinine

Authors and year of publication	Patient characteristics	Serotoninergic medication	Rasagiline dose	Other PD therapy	Clinical presentation of serotonin syndrome	Laboratory values	Treatment	Outcome
Duval et al. (2011) [9]	75-year-old woman with PD	Sertraline 100mg/day	1mg/day	Levodopa-carbidopa, entacapone, amantadine	Agitation, delusions, altered consciousness, diaphoresis, fever, unstable blood pressure, rigidity, hyperreflexia, tremors	CK: 1749U/L; CrCl: 30mL/min; BUN: 4.13mg/dL	Discontinuation of sertraline, rasagiline, amantadine; parenteral hydration	Full recovery within 3 days
Suphanklang et al. (2015) [10]	77-year-old man with PD	Escitalopram 20mg/day	1mg/day	Levodopa-carbidopa, entacapone, piribedil, pramiprexole	Agitation, confusion, behavioral change, hallucination, memory loss, fever, tremors	CK: 125700U/L; AST: 2361U/L; ALT: 504U/L; sCr: 8.9mg/dL	Discontinuation of escitalopram and rasagiline; benzodiazepines; hemodialysis	Recovery within 16 days; residual renal impairment
Hebant et al. (2016) [11]	72-year-old man with PD	Paroxetine 20mg/day	1mg/day	Levodopa-carbidopa, entacapone, rotigotine	Confusion, fever, diaphoresis, hyperventilation, tremors	WBC: 11000/mm^3^; CK: 1000IU/L	Discontinuation of paroxetine, rasagiline, rotigotine; benzodiazepines; parenteral hydration	Full recovery within a few days

The main differential diagnosis in our patient was MS. Malignant syndrome, similar to neuroleptic malignant syndrome (NMS), happens when there is sudden central dopamine deficiency. However, in contrast to NMS, MS happens when there is an abrupt reduction in dose or discontinuation of dopaminergic drugs [13].

Three common precipitants of MS in Parkinson’s disease are infection, dehydration, and surgery [14]. One possible explanation is that in these situations, patients have lower levels of compliance with their medication regimen. In the current medical literature, MS is also referred to as malignant syndrome in Parkinson’s disease, parkinsonism hyperpyrexia syndrome, or neuroleptic malignant-like syndrome [15].

The presentation of MS and SS can sometimes be almost indistinguishable. Most commonly, MS presents with fever, altered mental status, muscle hypertonia, and autonomic instability [13]. Our patient was treated with multiple dopaminergic drugs with very high doses for her Parkinson's disease. Furthermore, she developed a urinary tract infection. One probability can be that infection-associated anorexia leads to reduced compliance for her antiparkinson therapy [13], which could have precipitated MS. While there are several diagnostic criteria for NMS/MS [16], they are not frequently used in clinical practice. The key differentiating feature between SS and MS is the type of neuromuscular excitability. In SS there is hyperreflexia, clonus and tremor. In contrast, MS is characterized by rigidity (commonly referred to as ‘lead-pipe’) and hyporeflexia [2]. Clinical features of SS and MS are summarized in Table 3. The reason we included MS in our differential diagnosis, besides the possible dopaminergic withdrawal, is elevated CK as this finding is common in patients with NMS (more than 75%) [2]. Yet SS can cause rhabdomyolysis and a subsequent increase in serum CK values, especially in severe cases. Thus, laboratory values are not very useful when it comes to differentiating these disorders [2].

**Table 3:** Clinical features of serotonin syndrome and malignant syndrome

Features	Serotonin syndrome	Malignant syndrome
Causes	Serotoninergic drugs interaction	Withdrawal of dopaminergic drugs
Fever or hyperthermia	Present, often >38.0°C	Present, often >38.0°C
Mental status alteration	Confusion, agitation	Confusion, agitation
Autonomic dysfunction	Blood pressure fluctuation, tachycardia, diaphoresis, diarrhea, nausea, vomiting	Blood pressure fluctuation, tachycardia, diaphoresis, urinary incontinence
Neuromuscular hyperactivity	Myoclonus, hyperreflexia, tremor	Hypertonia, hyporeflexia, rigidity

It is important to differentiate between SS and MS, as the treatment approach depends on the correct diagnosis. While the mainstay of treatment for both conditions is supportive, there are some key differences. For SS, it is of utmost importance to stop all serotonergic medication [6]. We discontinued duloxetine and rasagiline in our patient immediately upon admission. In contrast, for suspected MS, dopaminergic medication should be re-initiated in Parkinson's patients experiencing possible withdrawal [13]. For that reason, we continued all the other antiparkinson medications on our patient's medication list. Next, the specific therapy for SS and MS also differs. Cyproheptadine is an off-label drug that works as a serotonin antagonist and is used as an antidote for SS [6]. However, multiple studies showed the poor efficacy of this medication [6]. On the other hand, dantrolene is a central and peripheral muscle relaxant widely used for patients with MS. Nevertheless, similarly to cyproheptadine, studies have failed to show its efficacy [17]. For these reasons, after discontinuing duloxetine and re-starting all her antiparkinson medication, we treated our patient only with supportive measures. 

## Conclusions

The purpose of this case report is to raise awareness of possible SS in patients taking MAO-B inhibitors such as rasagiline. As depression is not uncommon in Parkinson's disease, these patients are often treated with antidepressants. Thus, the combination of two drugs can precipitate SS. We expect large randomized controlled studies to verify the safety of the SSRI/SNRI-MAO B inhibitors combination in patients with Parkinson's disease. Finally, we intended to present the diagnostic troubles when it comes to differentiating between SS and MS, especially in patients with Parkinson's disease. This patient population often receives high doses of dopaminergic medication. Thus, even a slight dose reduction can lead to dopamine withdrawal and MS.
